# The Complex Role of C-Reactive Protein in Systemic Lupus Erythematosus

**DOI:** 10.3390/jcm10245837

**Published:** 2021-12-13

**Authors:** Helena Enocsson, Jesper Karlsson, Hai-Yun Li, Yi Wu, Irving Kushner, Jonas Wetterö, Christopher Sjöwall

**Affiliations:** 1 Department of Biomedical and Clinical Sciences, Division of Inflammation and Infection, Linköping University, SE-581 85 Linkoping, Sweden; helena.enocsson@liu.se (H.E.); jesper.karlsson@liu.se (J.K.); jonas.wettero@liu.se (J.W.); 2 MOE Key Laboratory of Environment and Genes Related to Diseases, School of Basic Medical Sciences, Xi’an Jiaotong University, West Yanta Road, Xi’an 710061, China; lihaiy@xjtu.edu.cn (H.-Y.L.); wuy@lzu.edu.cn (Y.W.); 3 The Affiliated Children’s Hospital of Xi’an Jiaotong University, West Yanta Road, Xi’an 710061, China; 4 Division of Rheumatology, Department of Medicine, Case Western Reserve University at MetroHealth Medical Center, 2500 MetroHealth Dr., Cleveland, OH 44109, USA; ixk2@case.edu

**Keywords:** acute-phase protein, autoimmunity, cardiovascular risk, C-reactive protein, inflammation, organ damage, systemic lupus erythematosus

## Abstract

C-reactive protein (CRP) is well-known as a sensitive albeit unspecific biomarker of inflammation. In most rheumatic conditions, the level of this evolutionarily highly conserved pattern recognition molecule conveys reliable information regarding the degree of ongoing inflammation, driven mainly by interleukin-6. However, the underlying causes of increased CRP levels are numerous, including both infections and malignancies. In addition, low to moderate increases in CRP predict subsequent cardiovascular events, often occurring years later, in patients with angina and in healthy individuals. However, autoimmune diseases characterized by the Type I interferon gene signature (e.g., systemic lupus erythematosus, primary Sjögren’s syndrome and inflammatory myopathies) represent exceptions to the general rule that the concentrations of CRP correlate with the extent and severity of inflammation. In fact, adequate levels of CRP can be beneficial in autoimmune conditions, in that they contribute to efficient clearance of cell remnants and immune complexes through complement activation/modulation, opsonization and phagocytosis. Furthermore, emerging data indicate that CRP constitutes an autoantigen in systemic lupus erythematosus. At the same time, the increased risks of cardiovascular and cerebrovascular diseases in patients diagnosed with systemic lupus erythematosus and rheumatoid arthritis are well-established, with significant impacts on quality of life, accrual of organ damage, and premature mortality. This review describes CRP-mediated biological effects and the regulation of CRP release in relation to aspects of cardiovascular disease and mechanisms of autoimmunity, with particular focus on systemic lupus erythematosus.

## 1. Introduction

Although more than 90 years have passed since the discovery of C-reactive protein (CRP) at The Rockefeller University, our current understanding of CRP is essentially based on the original observations made by William S. Tillett and Thomas Francis Jr. in the laboratory of Oswald T. Avery. They found that sera obtained from patients during the acute phase of pneumococcal pneumonia precipitated with the C-polysaccharide derived from the cell wall of the pneumococcus, and that this reaction diminished as the patients recovered [1,2]. This previously unknown C-reactive substance was later found to be a protein, and thus was named “C-reactive protein” [1,2]. The ligand to which CRP bound associated with teichoic acid and was identified in the 1970s as phosphorylcholine, which is abundant on the surfaces of microbes and apoptotic cells [3].

Today, we know that CRP is a highly conserved and ubiquitous protein in vertebrates and invertebrates [4]. In humans, CRP is a liver-derived acute-phase protein that consists of five identical 23-kDa globular subunits arranged in a pentameric structure with a discoid shape. In addition to the short pentraxins, CRP and serum amyloid P component (SAP), the pentraxin superfamily contains long pentraxins, i.e., neuronal pentraxin 1 (NPTX1), neuronal pentraxin 2 (NPTX2), neuronal pentraxin receptor (NPTXR), pentraxin 3 (PTX3), and pentraxin 4 (PTX4) [5].

The integrity of the native pentameric structure of CRP (pCRP) is dependent upon the presence of calcium ions. This structure is disrupted irreversibly into monomers under denaturing conditions, e.g., in an acidic microenvironment. Such CRP monomers (mCRP) appear to have distinct biological properties, which are often different from those of pCRP [6]. In addition, mCRP has been shown to act as an autoantigen in systemic lupus erythematosus (SLE), as well as in certain other diseases [7].

CRP is produced in large quantities by hepatocytes, mainly in response to the pro-inflammatory cytokine interleukin-6 (IL-6) [8]. The profound clinical interest in CRP arises from its use as a sensitive biomarker of ongoing bacterial infections, trauma, ischemic cardiovascular disease (CVD) and other inflammatory conditions, as well as its use as a crude discriminator of bacterial from viral infections, since bacterial infections typically yield higher levels of circulating CRP. However, in conditions characterized by the Type I interferon (IFN) gene signature (e.g., SLE, primary Sjögren’s syndrome and inflammatory myopathies), CRP appears to be an unreliable marker of inflammation, since the circulating levels of CRP can be modest—despite the presence of extensive inflammation, as evidenced by an increased level of IL-6 in the circulation [9,10]. Furthermore, several studies of cardiovascular and autoimmune diseases have highlighted the importance of the genetic regulation of CRP [11,12].

In parallel with the discovery that a low-level increase in CRP is a useful risk marker for cardiovascular events, substantial progress has been made over the last decades concerning the biological properties and physiological importance of CRP in both health and disease. This review summarizes recent discoveries related to CRP-mediated biological effects, as well as to the regulation of CRP release with respect to aspects of CVD and mechanisms of autoimmunity.

## 2. CRP as a Biomarker in Rheumatologic Diseases

CRP is the main biomarker of inflammation used in modern healthcare. In most laboratories in Europe, for routine detection of CRP, the cut-off defining an abnormal level is set at 5 or 10 mg/L. However, for estimation of CVD risk, a ‘high-sensitivity’ CRP assay is usually applied [13,14]. At Linköping University Hospital (Sweden), the lower limit of quantification for this high-sensitivity CRP assay is 0.15 mg/L.

Historically, CRP has not always been the most popular biomarker reflecting inflammation. Several other acute-phase proteins show different concentration pattern changes in the plasma over time; some of these increase (e.g., serum amyloid A) and some decrease (e.g., albumin) during the acute-phase response [15,16]. In rheumatology, the erythrocyte sedimentation rate (ESR), which is a reflector of ongoing inflammation, deserves special attention. However, whereas the kinetics of ESR is slightly different from that of CRP, it conveys different information and can be affected by various factors, such as the erythrocyte count and fibrinogen and immunoglobulin concentrations.

In the newest set of classification criteria for rheumatoid arthritis (RA), ‘abnormal CRP and/or ESR’ is regarded as a separate item together with joint involvement, presence of autoantibodies and duration of symptoms [17]. CRP levels >10 mg/L are frequently seen in untreated patients with recent-onset RA. Other types of arthritis show different tendencies to display abnormal CRP levels. During an attack of gout, the concentration of CRP can become impressively high, often arousing a suspicion of septic arthritis. In spondylo-arthritides, such as psoriatic arthritis (PsoA), high CRP levels are usually less common, although patients with involvement of large joints may constitute exceptions to this. Consequently, abnormal levels of acute-phase proteins were not included in the classification criteria for PsoA [18].

In giant cell arteritis (GCA), unexplained high levels of CRP and ESR, accompanied by unspecific symptoms such as weight-loss and headache, may lead to a correct diagnosis [19]. GCA may present with or without proximal muscular pain, referred to as polymyalgia rheumatica (PMR). Besides muscular involvement, the 2012 classification criteria for PMR require both age ≥50 years and abnormal CRP and/or ESR levels [20]. In cases of anti-neutrophil cytoplasm antibody (ANCA)-associated vasculitis, high levels of CRP elevation are almost ubiquitous and appear to be associated with a higher risk of renal involvement [21].

While CRP levels usually parallel disease activity in inflammatory states, it is widely accepted that CRP is an unreliable biomarker in active SLE. Still, substantial CRP responses are observed in subsets of patients with SLE with certain manifestations (e.g., serositis and polyarthritis) [10,22]. In similarity to trivial viral infections, wherein the CRP levels typically remain low, SLE may manifest as oral ulcers, pleuritis/pericarditis and leukopenia, all of which commonly affect patients with viral infections. Another feature shared by viral infections and systemic inflammatory conditions, such as SLE, primary Sjögren’s syndrome and inflammatory myopathies, is the activation of the Type I interferon system [23,24]. This will be discussed in depth below (Section 5).

Although CRP is a valuable biomarker in the clinical management of several rheumatic conditions, it must always be interpreted with caution and in the context of the symptoms presented by the patient. Several of the immunosuppressive agents used in rheumatology render the patients more prone to infections; this is particularly true for high doses of corticosteroids [25]. The risks for malignancies and paraneoplastic syndromes, which mimic rheumatic diseases, are important to consider, especially as the risks for certain cancers are increased in patients with rheumatic diseases [26,27]. Finally, some of the immunosuppressive drugs in use today directly affect the ability of the hepatocytes to produce adequate levels of CRP. The most obvious examples of this are the IL-6 receptor inhibitors tocilizumab and sarilumab, which are mainly used in cases of RA, systemic juvenile idiopathic arthritis and GCA [28]. Moreover, IL-6 signaling (and consequently CRP) may also be significantly negatively affected in patients who are receiving Janus kinase inhibitors and high doses of corticosteroids.

## 3. CRP as a Biomarker Indicating Increased Risks of Cerebrovascular and Cardiovascular Diseases

Based on the results of several prospective epidemiologic studies, CRP has emerged as one of the most powerful predictors of CVD in the general population [29]. In the ‘Fragmin during Instability in Coronary Artery Disease’ (FRISC) trial, which included almost 1000 patients with unstable coronary artery disease, the CRP levels were strongly associated with long-term risk of death from cardiac causes, independently of other established risk factors (i.e., hypertension, smoking, diabetes, dyslipidemia) [14]. Furthermore, CRP has been shown to contribute to several stages of atherogenesis, such as endothelial dysfunction, atherosclerotic plaque formation, plaque maturation, and plaque destabilization and eventual rupture [30].

Patients with RA, as well as those with SLE, have increased mortality compared to the general population [31,32]. Increased mortality from CVD has been reported in epidemiologic studies that have focused on RA [33]. In similarity to RA, the risk of CVD-related death is increased in SLE [31,34].

In prospective studies, the incidence rates of myocardial infarction and stroke in patients with SLE have been found to be high. The relative risk of myocardial infarction or stroke compared to the normal population is approximately 2–3 [35,36,37]. The highest relative risks have been reported for premenopausal women (8–50-fold higher risk), early in the course of SLE (<1 year after diagnosis, risk increased 4–10-fold), and in patients with renal involvement (4–18-fold higher risk) [38,39,40]. Other studies have focused on examining the incidence of CVD in patients with SLE compared to the expected CVD incidence, based on the presence of traditional risk factors. Even here, the incidence of CVD has been found to be considerably higher than expected [41,42]. In addition, risk of mortality post-myocardial infarction seems to be higher in patients with SLE than in the normal population, at least in the short term, while the long-term risk of mortality post-stroke is also increased [43,44].

Approximately 30% of patients with SLE display antiphospholipid antibodies (at least one of the following: anticardiolipin or anti-β2-glycoprotein-I antibodies, or a positive lupus anticoagulant test) and about 15–20% suffer from antiphospholipid syndrome (APS), which is characterized by an increased risk of thromboembolic disease and/or pregnancy morbidity. Ischemic stroke is the most common arterial manifestation of APS, while myocardial infarction is less common [45].

Whereas some studies have focused on CRP levels as a risk factor for future cardiovascular events in RA, studies of CRP levels in patients with SLE in relation to risks of CVD or stroke are scarce [46,47,48,49]. Statin therapy is likely to be safe and seems to result in significant reduction of plasma CRP concentrations in patients with SLE [50]. For patients with SLE, the Systemic Lupus International Collaborating Clinics/American College of Rheumatology (SLICC/ACR) Damage Index (SDI) constitutes a validated instrument to assess irreversible organ damage, including myocardial infarction and stroke [51]. We identified two studies in which CRP levels were analyzed in relation to accrual of damage. In the Hopkins Lupus cohort, Lee et al. showed that serum CRP levels (measured with the high-sensitivity technique) were independently associated with the total SDI score, although not specifically for myocardial infarction or stroke [52]. Our group has reported a similar association between CRP and global SDI [53]. Furthermore, in the SLICC cohort, we evaluated whether CRP could be predictive in terms of future damage accrual but obtained negative results [54].

## 4. Immunoregulatory Functions of CRP and other Pentraxins in SLE

The high accumulation of apoptotic cell debris and the formation of antinuclear antibodies (ANA), together with dysfunctional elimination of immune complexes are all key features of SLE pathogenesis [55]. In this context, it is of particular interest that CRP immune function can be viewed as a less specific albeit rapidly produced innate ancestor version of the phylogenetically more recent antibodies of adaptive immunity [56]. CRP is a pattern-recognition molecule of the innate immune system, and its binding to ligands such as surface-exposed phosphorylcholine on, for example, cellular debris can mediate direct prophagocytotic opsonization [57] and interactions with immunoglobulin receptors (Fc receptors) [56], as well as trigger ‘classical’ complement activation [58]. The latter promotes additional opsonization through subsequent covalent surface-binding of activated complement proteins.

Immune complex clearance is generally supported by efficient classical complement activation, and SLE pathogenesis is indeed intimately related to this activation pathway. Although homozygous complement deficiencies are extremely rare, they tell us a great deal about the normal physiological activities of the complement system in humans [59]. Homozygous genetic deficiencies in the initial proteins of the classical complement pathway (C1 proteins) are linked to a very high risk of developing SLE [60], and single nucleotide polymorphisms of the *CRP* gene are associated with ANA formation and SLE, possibly via the lowering of CRP levels [12].

In vertebrates, surface binding via the recognition face of the CRP molecule activates the calcium-dependent classical arm of the complement cascade by binding complement protein 1q (C1q) via its effector face [61,62]. C1q binding to the mCRP isoform has been demonstrated [63], and mCRP is capable of supporting complement-dependent phagocytosis and the oxidative burst in phagocytes [64]. Unlike immunoglobulin G-triggered classical activation, CRP-mediated initiation of the classical route typically does not proceed to the membrane-attack complex-forming ‘terminal’ stage of complement activation [65]. This is most likely due to direct interactions of CRP with inhibitory complement regulators. It is well-established that CRP can bind to the soluble complement inhibitor factor H without compromising its inhibitory function, thereby limiting the continued activation of complement via the convertases, by accelerating their decay [66,67,68,69] and by serving as co-factor for Factor I in cleaving surface-bound C3b [70,71]. In addition, surface-bound mCRP can bind Factor H and, thereby, modulate complement activation [72]. Anti-C1q autoantibodies are frequently detected in lupus nephritis (LN) [73,74] and it is possible that autoantibodies targeting other proteins linked to classical complement activation, e.g., CRP, could affect the complement-mediated clearance of cellular debris [75,76]. CRP (and/or PTX3), complement, and immunoglobulins may co-localize with electron-dense deposits in glomerular LN [77,78]. Furthermore, it is possible that pre-immunization with pentraxins, leading to the triggering of anti-PTX3 antibody development, prevents progression to LN [77]. Anti-CRP antibodies appear to target mainly the motifs of mCRP (further described in Section 7) and are typically associated with LN. The mCRP amino acids 35–47 have been reported to represent an autoantibody target motif that is especially prone to anti-CRP binding in LN. From the complement-immunomodulatory point-of-view, it is interesting to note that this epitope also facilitates factor H binding and activity—which could be reversed by anti-CRP antibodies [79]. In accordance with this, factor H levels are low in LN and factor H dysregulation and polymorphisms are associated with active nephritis [80,81]. Other members of the factor H family, i.e., factor H-related Proteins 1 and 5, have recently been shown to be capable of binding DNA and subsequently recruiting mCRP and enhancing complement activation [82]. In addition, factor H-related Protein 4 has been reported to bind pCRP [83,84].

Ligand-bound CRP on necrotic cells and/or otherwise immobilized CRP can recruit the classical pathway inhibitor C4-binding protein (C4bp) while retaining the complement-inhibitory activity of C4bp [85,86]. It is possible that this C4bp–CRP interaction limits CRP–C1q binding [85,86], and thereby subsequent classical activation. Classical complement activation triggered by ligand-bound CRP may be downregulated during substantial increases in the concentrations of CRP, presumably through humoral CRP–C1q consumption [63,87]. This could also be of pathophysiologic relevance in SLE, where the CRP levels can be low despite active inflammation. Related to the now well-established role of collectins in the ‘lectin’ activation pathway of complement, a potential role of CRP in conveying C1q-dependent complement activation by collectin Placenta 1 has been reported [88]. This is a topic that would be interesting to pursue further in relation to autoimmune diseases.

In similarity to IgG, surface-bound CRP (and other pentraxins) can bind directly to all Fcγ-receptors [89], potentially activating phagocytes and facilitating elimination via phagocytosis, which is highly relevant for waste disposal mechanisms. The low-affinity FcγRIIa (CD32) has emerged as the primary functional CRP receptor [56,89,90,91]. However, unlike IgG, the CRP–FcγRIIa interaction depends on the *R* allele of the receptor polymorphism at amino acid 131 [92]. Since SLE pathogenesis is linked to immune complex-induced production of IFN-α by plasmacytoid dendritic cells, it is highly interesting to note that FcγRIIa also mediates the initial internalization of immune complexes that prompts intracellular TLR activation and activation of IFN-α [93]. Considering the protective effects of CRP seen in animal models of lupus, it is tempting to speculate that CRP acts as a modulator of IFN-α production by altering the immune complex handling by plasma-cytoid dendritic cells. Accordingly, Mold and Du Clos reported that CRP indeed inhibits such immune complex-triggered activation of IFN-α, although the mechanism appeared to involve instead the endosomal processing of immune complexes [94]. Additional mechanistic studies on the CRP-mediated downregulation of immune complex-triggered IFN-α in SLE are highly warranted. Another intriguing finding that merits further attention is the potentially immunomodulatory effect of the CRP interaction with FcαRI, the IgA receptor [95].

## 5. Regulation of CRP Synthesis in SLE

Hepatocytes are considered the major source of CRP, although extrahepatic syntheses have been reported [96,97,98,99,100]. The *CRP* gene is located on chromosome 1q23.2 and hepatic production of CRP is mainly regulated at the transcriptional level, with IL-6 and IL-1β being the most important inducers [101,102]. IL-6 signaling in hepatocytes mediates the activation and CRP promoter-binding of signal inducer and activator of transcription 3 (STAT3) [102] and the CCAAT/enhancer binding protein β (C/EBPβ) [101,103]. In hepatic cell lines, the addition of IL-1β and subsequent NF-κB activation are usually required for *CRP* transcription, whereas in primary hepatocytes, IL-6 is sufficient for CRP production [101,104,105].

Although CRP is generally an excellent biomarker of inflammation and tissue damage due to its massive increase in level upon IL-6 induction, it is not useful in all inflammatory conditions. SLE represents an exception, in that the CRP levels rarely mirror the disease activity [15,106]. Inflammatory myopathies, primary Sjögren’s syndrome and systemic sclerosis are other diseases for which CRP is considered an unreliable marker for monitoring disease activity [107]. In addition, viral infections rarely exhibit a substantial rise in CRP levels [108].

The above-mentioned conditions all have in common the activation of Type I IFNs. The most widely studied Type I IFN is IFN-α, which comprises 12 subtypes. Apart from having a physiologic function in defense against viruses, IFN-α induces and maintains autoimmune pathology through facilitation of autoantibody production and many other functions, as reviewed elsewhere [109]. Receptors for Type I IFNs (IFN-α /β receptor; IFNAR) are ubiquitously expressed and mediate the activation of different STAT heterodimers and homodimers for the activation of antiviral, inflammatory and regulatory gene expression [110]. Already in 2008, Type I IFNs were highlighted as potential inhibitors of CRP production via their activation of STAT1, so as to counteract the STAT3 effects, and/or the activation of an inhibitory isoform of C/EBPβ [10]. Later, an inhibitory effect of IFN-α (all subtypes) on CRP transcription and production was indeed shown in a hepatic cell line and in primary hepatocytes, respectively [104]. Further in vivo studies of CRP levels and IFN-α levels in patients with SLE have lent support to the notion of a regulatory role for IFN-α in CRP production [54,111], although the exact intracellular pathways remain unknown.

**Figure 1:**
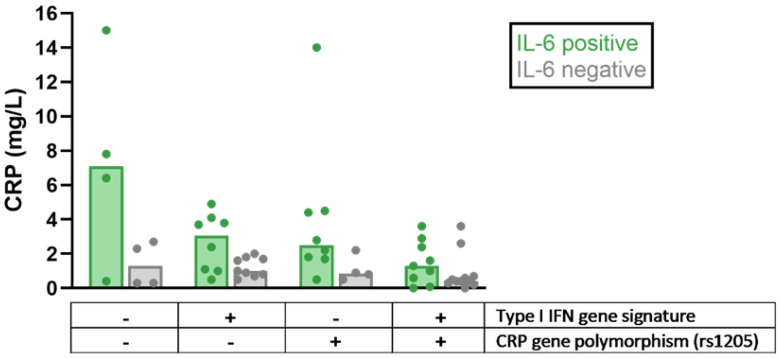
Differences in C-reactive protein (CRP) levels among patients with systemic lupus erythematosus (SLE) stratified based on the presence of detectable interleukin 6 (IL-6) levels, Type I interferon (IFN) gene signature, and CRP-lowering gene polymorphism (rs1205), respectively. All patients had a low disease activity but could be serologically active at sampling. Bars indicate median values. Dots represent individual values. Data shown in the figure were adopted from Enocsson et al. [115], with permission from Frontiers Media, 2021 (Creative Commons Attribution licence, version 4.0).

Polymorphisms of the *CRP* gene have been linked to differences in basal CRP levels and the risk of SLE and/or cardiovascular events [12,112,113,114]. One of these polymorphisms, rs1205, has been studied together with IL-6 and IFN-α with respect to the impact of these potential regulators of CRP levels in SLE, revealing lower CRP levels in patients with IFNα activation and/or the CRP-lowering polymorphism rs1205 (Figure 1). Thus, the relative lack of CRP response seen in viral infections and Type I IFN-driven autoimmune diseases can be attributed to an IFN-α-dependent downregulation of CRP transcription, as well as *CRP* gene polymorphisms, which are over-represented among patients with SLE [111,115].

## 6. Structural Isoforms of CRP with Distinctive Biologic Effects

As mentioned above, pCRP can under certain conditions dissociate irreversibly into the monomeric form (mCRP), which displays distinctly different conformational characteristics and antigenic epitopes [116,117]. Emerging data implicate mCRP as the main CRP isoform that regulates local inflammatory processes [118,119,120]. Furthermore, mCRP may bind to IgG-containing immune complexes and facilitate silent Fc receptor-mediated removal via the reticuloendothelial system and complement deficiencies may result immune complex deposition outside the reticuloendothelial system [121,122].

Cell death occurs during inflammation, and the damaged cell membrane in apoptosis or necrosis is the main target of CRP recognition [4]. Using electron microscopy, the detection of new epitopes of the antigen, and immunofluorescence colocalization, Ji et al. have shown that the binding of CRP to the damaged cell membrane induces rapid transformation to mCRP, and that this dissociation process is accompanied by significant enhancement of complement activation and cellular stimulation capacity [118]. Eisenhardt et al. obtained similar results with activated platelet membranes [123]. In addition, inflammatory conditions such as moderate acidification and oxidative stress also promote conformational switching from pCRP to mCRP.

During acute inflammatory cardiovascular events, such as thrombosis and myocardial infarction, the CRP levels increase rapidly, while activated platelets in blood vessels or cell necrosis caused by hypoxia in the heart provide abundant damaged membrane ligands for CRP dissociation, which leads to the accumulation of a large amount of ’active’ mCRP in the lesions within a short period of time [118,124]. This results in the excessive activation of neutrophils, platelets, monocytes and complement, thereby exacerbating the inflammation [118,123,125,126,127,128,129,130]. Furthermore, the conversion of pCRP to mCRP has been observed on microparticles in the blood obtained from patients who suffered myocardial infarctions, as well as on beta-amyloid plaques [131,132]. This process indicates a physiologic mechanism of CRP isomerization that is driven by the inflammatory microenvironment and, at the same time, supports the concept of mCRP occurring as a natural isomer of CRP and being involved in regulating inflammatory processes [120,133].

Most of the abovementioned dissociation scenarios for generating mCRP are specifically linked to inflammation. Thus, it is plausible that mCRP is generated predominately within the inflamed local tissue. Based on the strong proinflammatory activities of mCRP, we propose that, in addition to being an activating mechanism, the conversion of pCRP to mCRP serves as a buffering mechanism that localizes the proinflammatory actions to the site of the inflammation [118]. This mechanism could protect the body from systemic challenge in response to increased circulating levels of pCRP. It is worth noting that the bioactivities of mCRP largely overlap with, and occasionally exceed, those previously ascribed to pCRP. These bioactivities of mCRP include the activation of complement and the stimulation of endothelial cells, neutrophils and platelets, as well as its binding to ligands, e.g., LDL, C1q and factor H [63,72,85,126,128,134,135,136,137,138,139,140,141,142,143]. This raises the possibility that some of the reported actions of pCRP originate from mCRP formed during the purification process and/or storage.

The allosteric switch from pCRP, as a marker of inflammation, to functional mCRP that actually participates in the inflammatory process, enables this acute-phase protein to play active roles in a controlled manner under different pathophysiologic conditions. Thus, CRP can be regarded as a potential fine tuner of inflammation. Although there have been long-term debates about the biological significance of mCRP, recent studies have revealed the pathway of mCRP production, the regulatory effects of mCRP on innate and adaptive humoral immunity and inflammatory processes, and the presence of mCRP in focal tissues [63,118,123,126,128,134,135,136,137,138,142,144,145].

Interfering with the dissociation of CRP and the way in which mCRP exerts its biologic functions are candidate pathways towards designing treatment strategies for CVD. Since the specific contribution of mCRP depends on the inflammatory microenvironment, a clear understanding of the molecular mechanisms that act in different pathophysiologic conditions is a prerequisite for the design and selection of appropriate interventions. Several important issues remain to be resolved: (1) how to establish either direct or indirect detection methods that use mCRP as a disease marker; (2) how to establish an association between mCRP and disease processes; (3) how to describe the short-and long-term response profiles of different cell types to mCRP in a systematic way; and (4) identification of the receptor (s) that mediate the downstream effects of mCRP in lipid rafts.

## 7. Autoantibodies Directed against CRP in SLE and Related Conditions

Already in the mid-1980s, the presence of autoantibodies against CRP was described and linked to the debilitated ability of CRP to solubilize chromatin in a patient with SLE [146]. Subsequently, Bell et al. reported a high frequency of IgG antibodies to cryptic epitopes of CRP, first in patients suffering from the ‘autoimmune-like’ toxic oil syndrome and thereafter in patients with SLE [147,148]. Similarly, we have shown a prevalence of anti-CRP antibodies of approximately 40% in patients with SLE, with a distinct positive correlation between antibody occurrence/concentration and disease activity.

In our first study, we demonstrated that some patients with SLE were anti-CRP antibody positive on one occasion but negative on another occasion [149]. In succeeding investigations, we analyzed the antibody levels in consecutive samples from 10 well-charac-terized patients with SLE and showed that the levels of anti-CRP antibodies paralleled the clinical disease activity, usually with high levels of these antibodies appearing during disease flares [150]. In total, 70% of the patients were positive for anti-CRP antibodies on at least one occasion, and the levels correlated with disease activity assessed using the SLE disease activity index (SLEDAI).

Our findings were essentially confirmed by Rosenau and Schur, who demonstrated the presence of antibodies against CRP in the sera obtained from patients with different rheumatologic conditions, including SLE, where they observed an autoantibody frequency of 23% [151]. However, in our hands, sera from patients with RA or inflammatory bowel disease have consistently been negative in the anti-CRP assay, whereas a few additional patients with primary Sjögren’s syndrome and chronic hepatitis C infection tested positive [149,152]. Others have found anti-CRP antibodies in patients with tubulointerstitial nephritis and uveitis (TINU) syndrome [153]. Furthermore, Figueredo et al. have demonstrated the presence of anti-CRP antibodies in patients with SLE with or without APS; the anti-CRP-positive cases with SLE had lower C3 levels and were more likely to have anti-dsDNA and anticardiolipin antibodies as compared to the anti-CRP antibody-negative individuals. In addition, the frequency of LN was higher among the anti-CRP antibody positive SLE cases [154]. The biological properties of anti-CRP antibodies have also been investigated. Janko et al. demonstrated that anti-CRP—as well as anti-dsDNA-antibodies bind to apoptotic materials and, via clearance by macrophages, induce a pro-inflammatory cytokine response [75].

More recently, a large longitudinal study from Europe identified the presence of anti-CRP antibodies at the onset of LN as a strong risk factor for a composite outcome of non-response, renal flare, and end-stage renal disease after 2 years of standard LN treatment [155]. Analyses of the antigen specificity of the anti-CRP assay have revealed that autoantibodies to CRP in SLE are directed towards hidden epitopes, or neo-epitopes, of CRP (e.g., mCRP), and that immune complexes isolated from SLE sera do not induce false positive anti-CRP antibody test results [79,156,157]. Thus, in similarity to anti-C1q antibodies in SLE, reacting exclusively with an epitope that is exposed on structurally modified C1q [59,158], anti-CRP antibodies bind to mCRP on cells, as well as on tissues and in solution [76].

## 8. Conclusions

**Figure 2:**
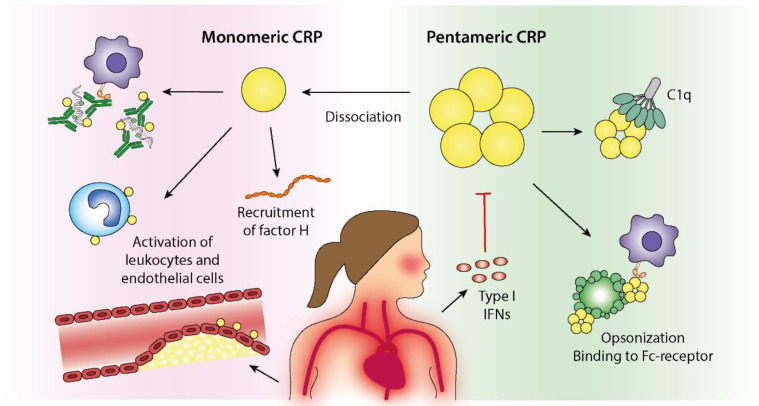
Immunoregulatory effects of pentameric C-reactive protein (pCRP) and monomeric CRP (mCRP) in the context of systemic lupus erythematosus (SLE) and cardiovascular disease. Dissociation of pCRP to mCRP will take place in inflammatory conditions and at cell surfaces and results in local immunoregulatory effects. The biological properties of mCRP partly overlaps the pCRP effects but is generally ascribed a more active and proinflammatory profile. CRP binds to and opsonizes dying cells and cell remnants, which facilitates phagocytosis via Fc-receptor binding. Furthermore, CRP activates classical complement activation via its binding to C1q, resulting in increased opsonization by C3b. Recruitment of Factor H will however limit progression of the complement cascade to membrane attack complex formation. Increased levels of CRP can therefore contribute to efficient clearance of potential autoantigens and thus, be beneficial in autoimmune conditions. The ability of CRP to facilitate immune complex elimination further implies a protective role of CRP in autoimmune diseases. However, increased Type I IFN activity, frequently observed in patients with SLE, inhibits CRP production, which theoretically could increase the autoantigen burden and disease activity. Proatherogenic and protrombotic effects of CRP are attributed to its stimulation of endothelial cells, neutrophils and platelets.

Even though almost a century has passed since the discovery of CRP, the biological effects of this highly conserved molecule are still poorly understood. Nonetheless, emerging data highlight the importance of structural isoforms of CRP and their associations with the complement system and CVD. As summarized in Figure 2, CRP plays a complex role in SLE—a disease in which CRP, in contrast to most other rheumatic conditions, constitutes an unreliable biomarker of inflammation. Recent data indicate that the combined effects of genetics and the Type I IFN signature are responsible for the dissociated correlation between CRP and IL-6 levels in patients with SLE. Given the potential activities of CRP in facilitating the removal of apoptotic debris and immune complexes, this may be of high relevance in terms of driving LN and the accrual of organ damage in SLE.
